# Centralized rapid genetic diagnosis of combined immunodeficiency in Japan

**DOI:** 10.1111/ped.70085

**Published:** 2025-06-03

**Authors:** Tamaki Kato, Yumi Ogura, Chikako Kamae, Tzu‐Wen Yeh, Tsubasa Okano, Junya Take, Kanako Mitsui‐Sekinaka, Kunihiko Moriya, Janine Reichenbach, Pandiarajan Vignesh, Amit Rawat, Surjit Singh, Masatoshi Takagi, Hirokazu Kanegane, Tomohiro Morio, Osamu Ohara, Kohsuke Imai, Shigeaki Nonoyama

**Affiliations:** ^1^ Department of Pediatrics National Defense Medical College Saitama Japan; ^2^ Department of Pediatrics and Developmental Biology Institute of Science Tokyo Tokyo Japan; ^3^ Department of Immunology University Children's Hospital Zurich Zurich Switzerland; ^4^ Allergy Immunology Unit, Advanced Pediatrics Center Postgraduate Institute of Medical Education and Research Chandigarh India; ^5^ Department of Child Health and Development, Graduate School of Medical and Dental Sciences Institute of Science Tokyo Tokyo Japan; ^6^ Department of Applied Genomics Kazusa DNA Research Institute Chiba Japan

**Keywords:** ataxia telangiectasia, combined immunodeficiency, next generation sequencer, severe combined immunodeficiency, T‐cell receptor excision circle

## Abstract

**Purpose:**

Severe combined immunodeficiency (SCID) is a pediatric emergency, and rapid genetic diagnosis is necessary for proper patient management, leading to successful stem cell transplantation and gene therapy. Ataxia telangiectasia (AT) requires early diagnosis to prevent infectious diseases and early detection of cancer. We aimed to diagnose patients with SCID/AT as quickly as possible and link them to the best treatments via the primary immunodeficiency database in Japan (PIDJ) network.

**Methods:**

For 111 patients with suspected combined immunodeficiency, including SCID/AT, we analyzed T‐cell receptor excision circle (TREC) and sequenced 29 causative genes of SCID, including ATM, by ion semiconductor sequencing using multiplex polymerase chain reaction amplicons. In some cases, DNA extracted from dried blood spots was used for the analysis.

**Results:**

Approximately 70.8% of 0–1‐year‐old patients and 26.5% of the patients >2 years old with low TREC were diagnosed genetically, including *ADA*, *ATM*, *IL2RG*, *IL7R*, *JAK3*, *RAG1*, *RAG2*, *DCLRE1C*, *NHEJ1*, and *LIG4*. However, only 6.9% of patients with normal TREC were genetically diagnosed (*STIM1* and *ATM*) in our panel. In Japan, all patients had been genetically diagnosed after infection or other life‐threatening conditions, and >80% of patients are linked to appropriate treatment after diagnosis.

**Conclusions:**

Target gene sequencing, including SCID and AT genes, was useful for the diagnosis of patients with combined immunodeficiency with low TREC and to lead them to prompt treatment and better prognosis.

## INTRODUCTION

Severe combined immunodeficiency (SCID) is a human inborn error of immunity (IEI) associated with absent or low levels of serum immunoglobulin and marked abnormalities in the number and/or function of T lymphocytes.^1^ SCID is a serious genetic disease in immunocompromised patients that occurs in the early postnatal period. To date, >20 causative genes of SCID and >40 causative genes of CID, which are milder than SCID, have been identified.^2^ These milder CIDs were defined as leaky SCID^3^ or late‐onset combined immunodeficiency (LOCID)^4^ as the patients with hypomorphic *IL2RG* mutations,^5^ LIG4^6^, ^7^ or RAG1.^8^ SCID becomes fatal within 2 years of age unless hematopoietic stem cell transplantation (HSCT), enzyme replacement therapy, or gene therapy is performed. Early diagnosis before infection is important because the prognosis is poor if patients suffer from an infection. Additionally, some types of SCID are highly radiosensitive; thus, it is important to identify the causative gene for the appropriate choice of conditioning regimen for stem cell transplantation and avoid DNA‐damaging radiological tests.^9^

Ataxia telangiectasia (AT) is an autosomal recessive disorder with progressive multiorgan disorder that manifests at the beginning of walking, such as progressive ataxia, immunodeficiency, frequent tumor development, endocrine disorders, radiosensitivity, and telangiectasia. The frequency of AT is 1 in 40,000–100,000 individuals, and the causative gene is *ATM*. The *ATM* gene is a large, located on chromosome 11q22.3, with a length of approximately 150 Kbp and 66 exons. Immunodeficiency in AT is characterized by a decrease in the numbers of CD3 + CD4 + T, CD20 + B, and CD4 + CD45RA + naïve T cells. The T‐cell receptor excision circles (TREC) decrease in almost all cases, reflecting a decrease in T cells. Hypogammaglobulinemia and low IgA levels have also been recognized, and respiratory infections are important. Early diagnosis is necessary for the prevention of infectious diseases and the detection of cancer.^10^

T‐cell receptor excision circles (TREC) are circular DNA generated in the process of α chain *VDJ* gene rearrangement. It is not replicated during T‐cell differentiation/proliferation and exist stably; therefore, they can be used as markers for T‐cell neogenesis. We and others have developed and reported a method for the absolute quantification of TREC using real‐time polymerase chain reaction (PCR) and its application in newborn screening (NBS) for SCID.^11^, ^12^ In 2008, the NBS of SCID using TREC analysis started and was extended in the United States.^13^, ^14^, ^15^ Pilot studies have also started in Europe^16^, ^17^, ^18^, ^19^ and Asian countries^20^ including Japan.^21^, ^22^

There are >20 causative genes related to SCID, and the conventional Sanger sequencing method is labor‐intensive and time‐consuming. *ATM*, the causative gene of AT, is a large gene with similar problems.^10^

For the rapid diagnosis of CID, including SCID, LOCID, and AT with low TREC, our department has developed a sequencing panel containing SCID‐related genes and *ATM* and has been clinically applied since 2014. Such trends have been reported both domestically and internationally for targeted resequencing using primary immunodeficiency (PID) and SCID next generation sequencer (NGS).^23^, ^24^

Newborn screening for SCID using TREC is performed worldwide.^22^ Especially in the United States, 90% of patients with SCID diagnosed in 2016 were found in NBS, and 10% were found in infectious diseases or family history.^15^ In the United States and Canada, 59% of 100 post‐transplant cases, such as SCID, leaky SCID, and Omenn syndrome, who underwent HSCT between 2010 and 2014 were diagnosed using NBS or family history. The overall survival rate at two years post‐transplantation was 90% compared to 81% in patients with active infections.^25^ Therefore, it is important to make a genetic diagnosis as early as possible and start treatment before infection occurs to improve patient prognosis. For rapid genetic analysis, we established the Primary Immunodeficiency Database in Japan (PIDJ), which is a diagnostic center for patients with PID and contains the clinical symptoms and results of genetic analysis of patients with PID in Japan.^26^

In this study, we quantified TREC in specimens of suspected patients with SCID, related diseases, or AT based on clinical symptoms, blood tests, and imaging tests. Genomic DNA was extracted from peripheral blood (PB) or from three punches of 3 mm in diameter using dried blood spots (DBS) for sequencing analysis, including copy number variant (CNV). Additionally, we analyzed the clinical course after sequencing analysis.

## MATERIALS AND METHODS

### Patients

A total of 111 patients (103 with clinically suspected SCID/CID and eight with suspected AT) were included in our study. Most patients are triggered by infections, which have led to the development of our system. Other triggers are life‐threatening symptoms, such as hemophagocytic lymphohistiocytosis (HLH), liver failure, and severe intestinal symptoms. Patients were recruited from the PIDJ network (*n* = 45),^26^ Postgraduate Institute of Medical Education and Research, Chandigarh, India (*n* = 62),^27^ and Universitäts‐Kinderspital Zürich, Zürich, Switzerland (*n* = 4) between December 2014 and December 2017. Sixty‐eight patients were males (61.3%) and 43 were females (38.7%). Seventy‐five patients were aged <2 years (67.6%), and 36 were aged >2 years (32.4%). Some of the patients have been previously used in a published study.^5^, ^6^, ^7^, ^8^, ^28^ All patients with AT were from India. Patients in whom the attending physician suspected immunodeficiency due to repeated infections and other symptoms were included in this study. The possibility that different attending physicians may differ in their assessment cannot be ruled out. Patients' information, including age at diagnosis, sex, details of infection, autoimmune disease, HSCT, prognosis, white blood cell count, lymphocyte count, serum immunoglobulin levels, lymphocyte surface antigen analysis (CD3, CD4, CD8, CD4/CD45RA, CD4/CD45RO, CD19, CD16/56), and thymus findings of echo or X‐ray, etc., were obtained.

The study protocol was approved by the Institutional Review Board of National Defense Medical College (approval number: 566 and 1275). Written informed consent was obtained from the patients and their parents in accordance with the Declaration of Helsinki.

### DNA extraction

Genomic DNA was extracted from 200 μL of PB or three punches of 3 mm in diameter from DBS using the QIAamp DNA Mini Kit or QIAcube (Qiagen, Hilden, Germany). Additionally, gDNA concentration was measured using BioDrop μLite (BioDrop UK Ltd., Cambridge, UK). We previously analyzed 133 control DBS samples and demonstrated that the mean amount of DNA extracted from DBS was 404 ng (range: 200–740 ng) (unpublished data). These DNA amounts were sufficient for analysis, as only 50 ng of DNA was required.

### TREC analysis

T‐cell receptor excision circle was quantified using DNA samples, as previously described.^12^ When TREC is <100 copies/μL of blood, it is defined as low, and ≥100 is defined as normal.

### *SCID/ATM* gene sequencing method

Polymerase chain reaction primer panels were designed to cover all exons and UTRs ± 5 bp of 28 *SCID* genes (causative genes of combined immunodeficiencies in the International Union of Immunological Societies classification of PID in 2011^29^) (*ADA*, *AK2*, *CD3D*, *CD3E*, *CD3G*, *CD3Z*, *CD8A*, *CORO1A*, *CRACM1 DCLRE1C*, *FOXN1*, *IL2RG*, *IL7R*, *JAK3*, *LCK*, *LIG4*, *MAGT1*, *NHEJ1*, *PNP*, *PRKDC*, *PTPRC*, *RAC2*, *RAG1*, *RAG2*, *RMRP*, *STAT5B*, *STIM1*, and *ZAP70*) and *ATM* using Ion AmpliSeq designer software v2.2.1 following the guide on the website (Thermo Fisher Scientific, IL, USA) (Table S1). All 29 genes exhibited X‐linked recessive or autosomal recessive inheritance. The design rates (in silico coverage of target sequences by multiplex PCR) were 100% for all 29 genes (Table S2).

Extracted DNA was amplified by multiplex PCR using designed 666 primer pairs. Barcode adapters were added for proband indexing to increase efficiency and were subsequently subjected to emulsion PCR on the Ion One Touch 2 system. The resulting enriched Ion Sphere particles were loaded and sequenced using an Ion PGM system (Thermo Fisher Scientific). Using this method, the final identification of the causative gene can be achieved within 2 days.

### Mutation analysis

The obtained data were analyzed using Ion Reporter software (Thermo Fisher Scientific), and mutations, such as single nucleotide variants (SNV) and small indels, were obtained. Candidate genes were narrowed down in the following order: (1) Minor Allele Frequency (MAF) <0.01; (2) non‐synonymous mutations that cause amino acid changes or mutations that cause splice changes; and (3) homozygous, compound heterozygous, and hemizygous mutations. Additionally, variants repeatedly detected in this system were excluded from the target as in‐house SNVs. Copy number variant analysis was performed. Polymerase chain reaction and electrophoresis were performed to confirm the deletions identified by the CNV analysis. The mutations that were finally identified were searched for in mutations registered as a cause of disease in the Human Gene Mutation Database (Qiagen, Hilden, Germany). If the variants had not been reported, we applied them to in silico pathogenicity prediction tools, including Mutation @ A Glance (https://mutation.nagahama‐i‐bio.ac.jp) and Mutation Taster (http://www.mutationtaster.org).

### Whole‐exome sequencing

Fourteen patients, whose causative genes could not be identified by targeted NGS sequence, were analyzed by whole‐exome sequencing.

### History of infection and clinical outcome of Japanese patients

Seventeen Japanese patients genetically diagnosed by our system were followed up about infection history, sites of infection, pathogenic microorganisms, and post‐diagnosis treatment methods.

## RESULTS

### Patients

**FIGURE 1:**
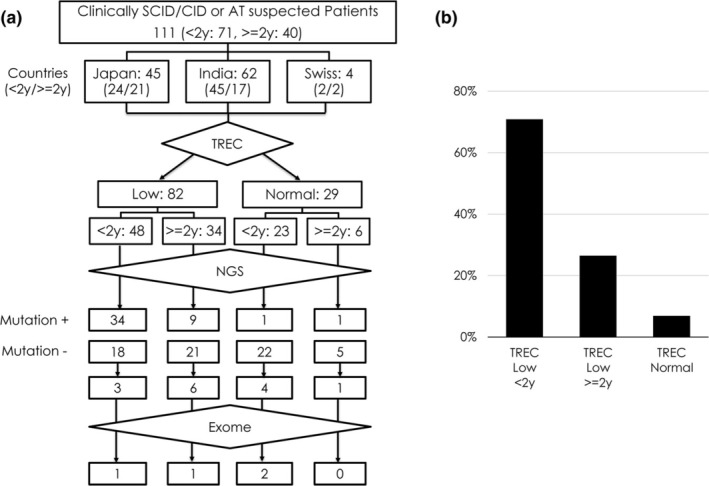
(a) Results of analysis of Clinically SCID/CID suspected Patients in our system. (b) Gene mutation detection rate.

A total of 111 patients were included in this study (103 clinically suspected patients with SCID/CID and eight with suspected AT) (Figure 1a). There were 45 patients (aged <2 years: 24, ≥2 years: 21) from Japan, 62 (aged <2 years: 45, ≥2 years: 17) from India, and 4 (aged <2 years: 2, ≥2 years: 2) from Switzerland. T‐cell receptor excision circle was low in 82 patients and normal in 29. Among the patients with low TREC, 48 were aged <2 years and 34 were ≥2 years. Among the patients with normal TREC, 23 were aged <2 years and 6 were aged ≥2 years (Figure 1a).

Ninety‐four patients had a history of infectious diseases. Two patients underwent HSCT prior to the genetic diagnosis. Mortality occurred in 51 patients, of whom 39 were from India. There were eight suspected AT patients, and increased α‐fetoprotein was observed in five patients. All eight patients were >2 years old and showed low TREC.

### Genetic diagnosis by target NGS

Using DNA from the PB or DBS of the patients, we performed multiplex PCR of the exon region and sequenced the gene panel using the Ion PGM system. In total, causative genes were identified in 49 of the 111 patients (44.1%). Forty‐five patients were identified by targeted NGS (41.0%), and four were diagnosed by whole‐exome sequencing (*EXTL3*, *CD40L*, *CXCR4*, and *CIITA*) after targeted NGS.^28^ If the patients were with low TREC and aged <2 years, the causative genes were identified in 34 (70.8%) of 48 patients. The causative genes are *ADA*, *IL2RG*, *IL7R*, *JAK3*, *RAG1*, *RAG2, DCLRE1C*, *NHEJ1*, and *LIG4*. If the patients were with low TREC and aged ≥2 years, the causative genes were identified in nine of 34 patients (26.5%). The causative genes identified were *IL2RG*, *LIG4*, and *ATM*. Causative genes were identified in two patients with normal TREC (*STIM1* and *ATM*) (Figure 1 and Table S3).

### History of infection and clinical outcome of Japanese patients

**FIGURE 2:**
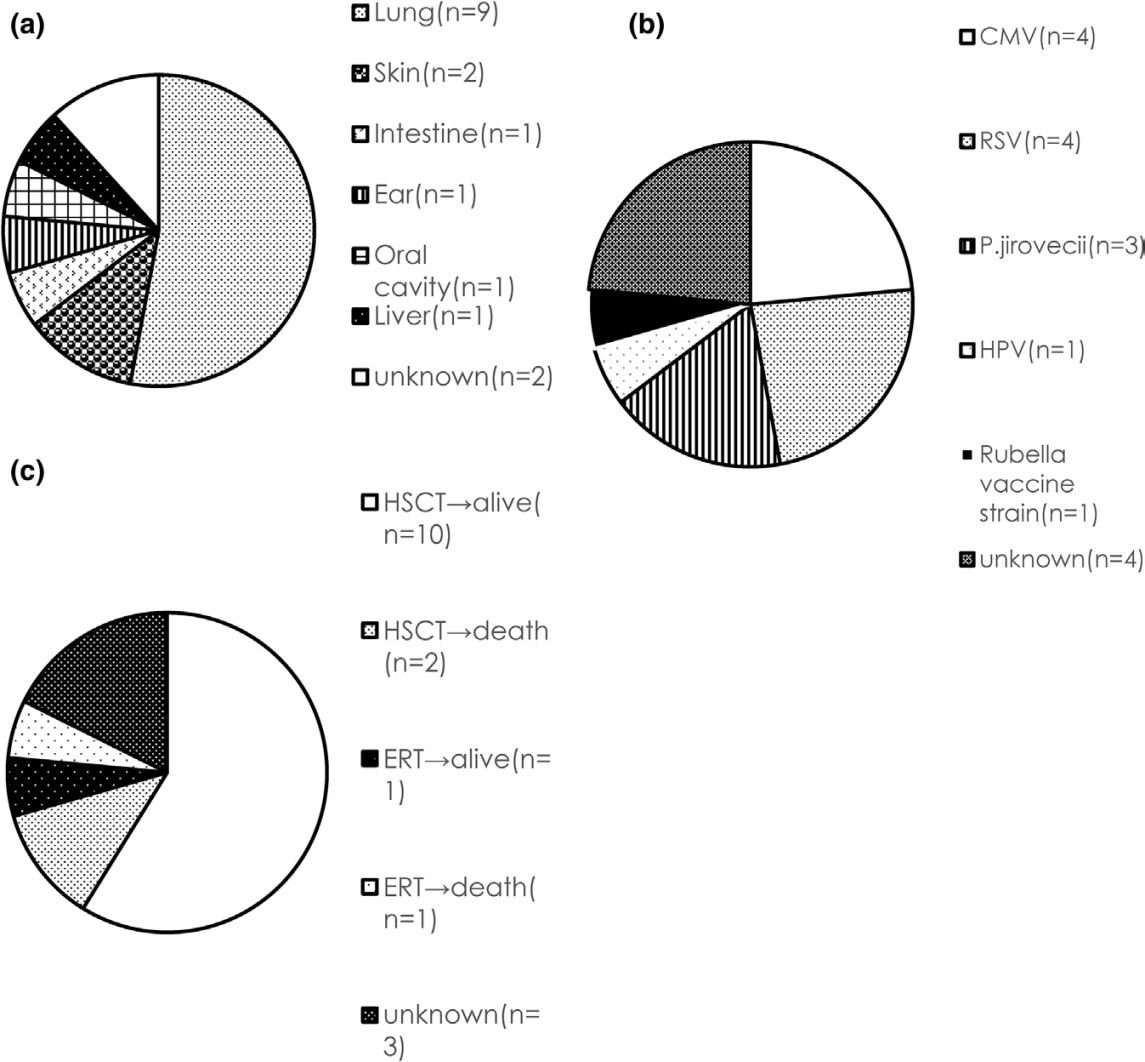
(a) Infection site of genetically diagnosed Japanese SCID patients (*n* = 17). (b) Pathogenic microorganisms of genetically diagnosed Japanese SCID patients (*n* = 17). (c) Treatment of genetically diagnosed Japanese SCID patients (*n* = 17).

Most of the Japanese patients with suspected SCID/CID were having infections (93.3%). The remaining three patients were suspected to have immune dysregulation. Sites of infection were as follows: nine patients had infections in the lungs (52.9%), 2 in the skin (11.8%), one in the intestines (5.9%), 1 in the ears (5.9%), and one in the oral cavity (5.9%) (Figure 2a). Causative microorganisms were as follows: cytomegalovirus (CMV), four (23.5%) patients; respiratory syncytial virus (RSV), four (23.5%); rubella vaccine strain, one (5.9%); and *Pneumocystis jirovecii*, three (17.6%) (Figure 2b).

Fourteen of 17 patients (82.4%) were linked to appropriate treatment. Twelve patients received HSCT, and 10 survived (81.8%), and two patients with *ADA*‐SCID received enzyme replacement therapy, and 1 survived (Figure 2c).

The following is a description of cases in which details of the course of events after the genetic diagnosis are available.

The patient, SCID3 having *ADA* mutations (Table S3) presented with lung and liver abscesses and CMV infection. At the time of diagnosis, enzyme replacement and infection treatment were performed immediately, but the infection was out of control, and the patient died of pulmonary hemorrhage.

The patient, SCID6 having a *DCLRE1C* mutation (Table S3) presented with CMV infection, pulmonary aspergillosis, and HLH. HSCT was performed after infection control; however, HLH recurred, and the patient died.

The patient, SCID11, having an *IL2RG* gene mutation (Table S3) showed active CMV infection at HSCT; therefore, PB stem cell transplantation was performed from an HLA1 locus‐mismatched father without pretreatment. Only T cells were engrafted, and CMV disappeared; however, diarrhea and fever appeared 1 year after transplantation, and norovirus could not be eliminated from the stool for >1 month. Flow cytometric analysis revealed T‐cell receptor (TCR) dysfunction due to TCRVβ bias; therefore, after pretreatment of the host, retransplantation was performed from the same donor, infection control was successful, and the patient was still alive.

The patient, SCID22, having an *IL2RG* gene mutation (Table S3), had pneumocystis pneumonia and pulmonary aspergillosis prior to HSCT. The HSCT was performed for infection control, but the infection recurred early after transplantation, and the patient died of respiratory and multiple organ failures.

Most foreign patients were from India except for four patients from Switzerland. Of the 12 patients in which the causative gene was confirmed, only three survived at the time of analysis, five died, and four were unknown as to whether they were alive or dead.^27^

## DISCUSSION

We have established a genetic diagnostic system for CID, including SCID, LOCID, and AT. Using this system, genetic analysis was centralized, and rapid genetic analysis was performed in Japan. The causative genes were identified in 41.0% (45/111) of the patients analyzed. In patients with low TREC, the percentage of causative gene identification was increased to 52.4% (43/82). The percentage of causative gene identification was higher in children aged <2 years (70.8%, 34/48) than in those aged ≥2 years (26.5%, 9/34) (Figure 1). These results indicate that our system is the most useful for identifying causative genes if the patient's TREC is low and the patient is aged <2 years. In contrast, if TREC were normal, the percentage of gene mutation identification was decreased to 6.9% (2/29). In two patients with normal TREC, we identified the causative genes using exome analysis after targeted NGS. Recently, we reported that 36 of 136 (26.5%) identified an IEI‐causing gene as a result of performing whole‐exome sequencing on patients with IEI.^29^ If a patient's TREC is normal and targeted NGS cannot identify the causative gene, it might be better to consider whole‐exome sequencing.

**FIGURE 3:**
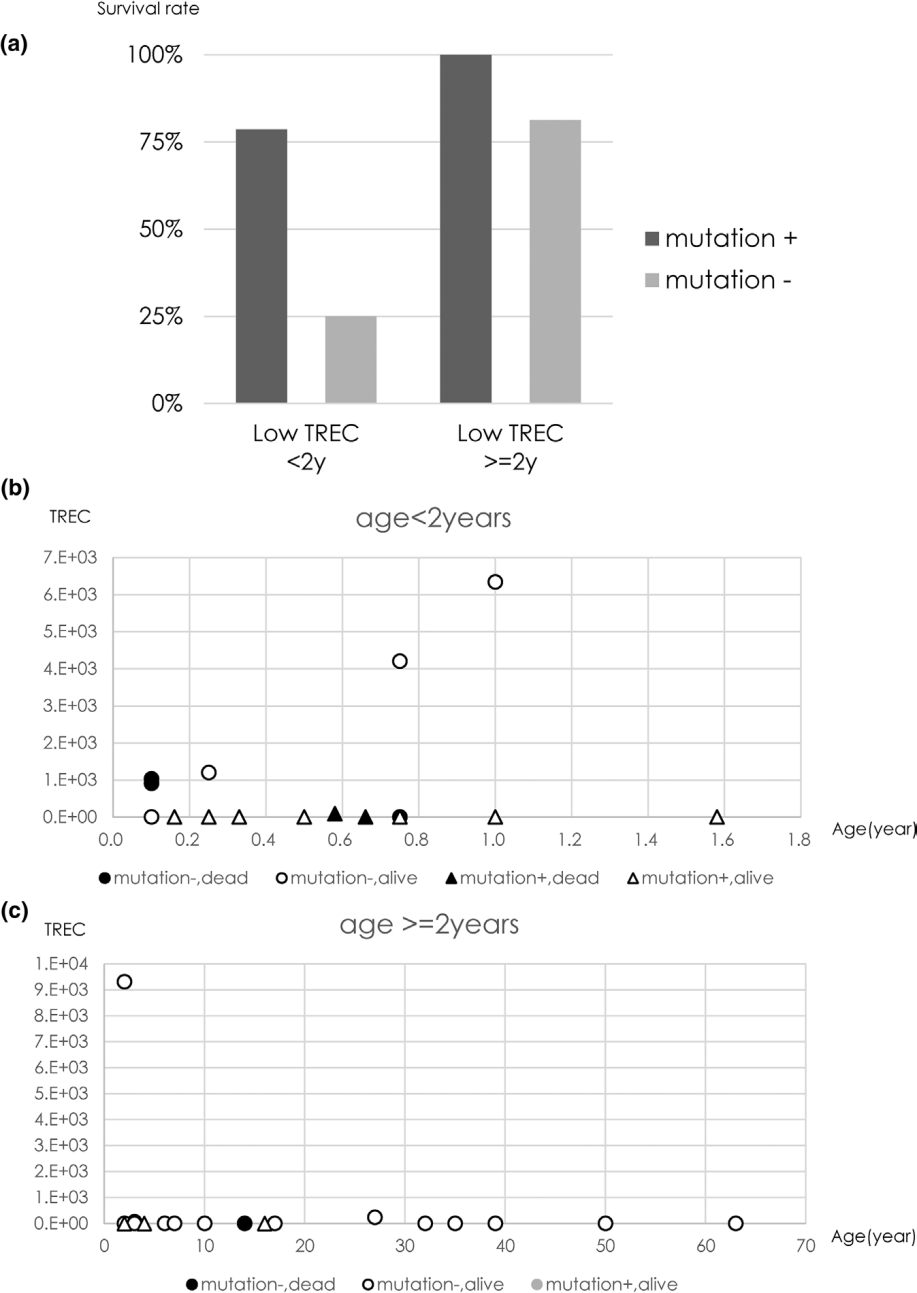
(a) Survival rate of SCID/CID clinically suspected Japanese Patients in our system. (b) Age, TREC, genetic diagnosis and survival results (<2 years). (c) Age, TREC, genetic diagnosis, and survival results (≥2 years).

In Japanese patients with low TREC, 11 of 14 (78.6%) genetically diagnosed at <2 years of age survived, and 1 of 5 (20%) cases not genetically diagnosed at <2 years of age survived. Three of 3 (100%) patients genetically diagnosed at ≥2 years survived, and 13 of 16 (81.3%) not genetically diagnosed at ≥2 years of age survived (Table 1 and Figure 3). Further case series are required in the future to find if the genetic diagnosis can contribute to improving the prognosis or the quality of life of the patients with low TREC levels. If a genetic diagnosis is made, patients can be treated with HSCT without delay, resulting in an improved prognosis.

**TABLE 1:** Results of analysis and survival of Clinically SCID/CID or AT suspected Japanese Patients in our system.

Low TREC	<2 years	18	Mutation +	14	Survival +	10
					Survival −	4
			Mutation −	4	Survival +	2
					Survival −	2
	≥2 years	18	Mutation +	3	Survival +	3
					Survival −	0
			Mutation −	15	Survival +	12
					Survival −	3
Normal TREC	<2 years	6	Mutation +	2	Survival +	1
					Survival −	1
			Mutation −	4	Survival +	2
					Survival −	2
	≥2 years	3	Mutation +	0	Survival +	0
					Survival −	0
			Mutation −	3	Survival +	3
					Survival −	0

Samples of gDNA are easily transported because they are stable at room temperature. Recently, X‐linked agammaglobulinemia was diagnosed with DNA derived from DBS shipped inexpensively by airmail across continents, by Sanger sequencing overseas.^30^ We also confirmed that our system could perform gene analysis with DNA from DBS of sufficient quality for NGS, and we believe that this would enhance the usefulness of our system.

In one patient, a mutation in *STIM1* was identified in a patient with SCID and normal TREC. Loss‐of‐function mutations in the *STIM1* gene are known to cause T+B+NK+SCID in normal T, B, and NK cells.^31^ Our system is useful for identifying SCID caused by *STIM1* mutations in normal TREC.

Mutations in the *ATM* gene were identified in six of the eight AT‐suspected patients with low TREC. Some AT patients are TREC‐negative even in the newborn period, as reported previously.^32^, ^33^, ^34^ Thus, we included the primers and probes for *ATM* in our CID panel to diagnose AT for TREC‐negative NBS‐positive newborns. Early diagnosis of AT may contribute to improving prognosis and quality of life because of the introduction of prophylactic treatment for infection, initiation of immunoglobulin substitution, and rehabilitation for speech and swallowing disorders without delay.

Most of the analyzed patients were diagnosed as a result of infection, and all were severe and intractable, making treatment difficult. Lung infections accounted for more than half of the infections among the patients with SCID in Japan surveyed in this study. The causative pathogens were CMV, RSV, and *P. jirovecii*, which together accounted for more than half of the patients. Regarding RSV, palivizumab (anti‐RSV antibody) administration can prevent the disease from becoming more severe; since there is insurance coverage for children with SCID below 24 months of age in Japan, it is better to diagnose and administer palivizumab before infection. Prophylactic use of trimethoprim‐sulfamethoxazole and antifungal medicines should be initiated to prevent infection with *P. jirovecii* and fungi. Because SCID is fatal due to infection, it is important to diagnose it as early as possible to prevent infection.

In Japan, most patients with SCID occur after infectious diseases are treated with HSCT at the time of the study period. The prognosis of HSCT in Japan was worse than that in countries that perform NBS for SCID using TREC analysis.^25^, ^33^ However, the situation is expected to change as NBS for SCID using TREC analysis has become more widespread in Japan over the past few years.^35^

All but four overseas patients were referred from India. Most patients in India died before a diagnosis was confirmed, probably because of a lack of recognition and facilities that analyze SCID in India. Centers for genetic analysis and HSCT have been established in India, and a summary of their work has recently been published.^27^, ^36^

Our system is useful for the rapid genetic diagnosis of patients with clinically suspected SCID/CID. A rapid and definitive diagnosis will result in appropriate treatment and a good prognosis. Rapid genetic analyses will become more important after the start of newborn screening for PID in India and Japan.

## AUTHOR CONTRIBUTIONS

T.K., Y.O., C.K., K.I., and S.N. contributed to the study's conception and design. All authors read and approved the final manuscript.

## CONFLICT OF INTEREST STATEMENT

The authors declare no conflict of interest.

## Supporting information


Table S1.

Table S2.

Table S3.
