# Supplementary material for: Centralized rapid genetic diagnosis of combined immunodeficiency in Japan
Source: Pediatr Int. 2025 Jun 3;67(1):e70085. doi: 10.1111/ped.70085 (PMC12130913; doi:10.1111/ped.70085)
Supplement: Supplementary file 2 — Table S2. [file PED-67-e70085-s001.docx]

Table S2

Design of SCID and AT panel primers

| **Nr.** | **Gene** | **Amplicons, n** | **Target, bp** | **Design rate** |
| --- | --- | --- | --- | --- |
| **1** | ***ADA*** | **16** | **1224** | **100** |
| **2** | ***AK2*** | **9** | **1091** | **100** |
| **3** | ***CD3*ζ** | **8** | **627** | **100** |
| **4** | ***CD3δ*** | **7** | **571** | **100** |
| **5** | ***CD3ε*** | **9** | **712** | **100** |
| **6** | ***CD3γ*** | **7** | **615** | **100** |
| **7** | ***CD8A*** | **10** | **774** | **100** |
| **8** | ***CORO1A*** | **16** | **1496** | **100** |
| **9** | ***CRACM1 - ORAI1*** | **11** | **934** | **100** |
| **10** | ***DCLRE1C - Artemis*** | **23** | **2274** | **100** |
| **11** | ***FOXN1*** | **17** | **2035** | **100** |
| **12** | ***IL2RG*** | **9** | **1198** | **100** |
| **13** | ***IL7R*** | **11** | **1468** | **100** |
| **14** | ***JAK3*** | **40** | **3628** | **100** |
| **15** | ***LCK*** | **21** | **1662** | **100** |
| **16** | ***LIG4*** | **16** | **2747** | **100** |
| **17** | ***MAGT1*** | **15** | **1214** | **100** |
| **18** | ***NHEJ1- Cernunnos*** | **9** | **977** | **100** |
| **19** | ***PNP*** | **9** | **936** | **100** |
| **20** | ***PRKDC*** | **140** | **13332** | **100** |
| **21** | ***PTPRC- CD45*** | **53** | **4316** | **100** |
| **22** | ***RAC2*** | **9** | **645** | **100** |
| **23** | ***RAG1*** | **15** | **3143** | **100** |
| **24** | ***RAG2*** | **9** | **1595** | **100** |
| **25** | ***RMRP*** | **3** | **278** | **100** |
| **26** | ***STAT5B*** | **26** | **2562** | **100** |
| **27** | ***STIM1*** | **17** | **2190** | **100** |
| **28** | ***ZAP70*** | **22** | **2164** | **100** |
| **29** | ***ATM*** | **109** | **9853** | **100** |
|  | **total** | **666** | **66261** | **100** |

The PCR primers amplify total 666 amplicons and 66,261bp. The Design rates are 100 % in all 29 genes.

Amplicons: numbers of amplicons

bp: numbers of base pairs

Design rate: in silico coverage of target sequences by multiplex PCR
